# 892. Epidemiology, clinical features, and outcomes of Rhinovirus/Enterovirus in older adults hospitalized with acute respiratory infections and those with CHF and COPD exacerbations

**DOI:** 10.1093/ofid/ofad500.937

**Published:** 2023-11-27

**Authors:** Gabby Ess, Ashley Tippett, Luis W Salazar, Olivia Reese, Caroline R Ciric, Laila Hussaini, Elizabeth Begier, Robin Hubler, Qing Liu, Bradford D Gessner, Benjamin Lopman, Satoshi Kamidani, Nadine Rouphael, Evan J Anderson, Christina A Rostad

**Affiliations:** Emory University School of Medicine, Atlanta, Georgia; Emory University, Atlanta, Georgia; Emory University, Atlanta, Georgia; Emory University, Atlanta, Georgia; Emory University, Atlanta, Georgia; Emory Univeristy, Atlanta, Georgia; Pfizer Vaccines, Dublin, Dublin, Ireland; Pfizer Inc., Collegeville, Pennsylvania; Pfizer Inc., Collegeville, Pennsylvania; Pfizer Biopharma Group, Collegeville, Pennsylvania; Rollins School of Public Health | Emory University, Atlanta, Georgia; Emory University School of Medicine and Children's Healthcare of Atlanta, Atlanta, Georgia; Emory University School of Medicine, Atlanta, Georgia; Moderna, Inc., Atlanta, Georgia; Emory University School of Medicine and Children's Healthcare of Atlanta, Atlanta, Georgia

## Abstract

**Background:**

While Rhinovirus/Enterovirus (RV/EV) infections are common, the clinical characteristics of infections in hospitalized adults are not fully understood.

**Methods:**

Adults ≥ 50 years of age hospitalized for Acute Respiratory Infections (ARI) or exacerbations of CHF or COPD in two hospitals in Atlanta, GA during the 2018-2019 and 2019-2020 respiratory seasons were offered enrollment. Following informed consent, participants were tested via BioFire® FilmArray® respiratory panels of nasopharyngeal and oropharyngeal swabs (combined), and standard-of-care molecular testing results were also recorded. Subjects were considered positive for RV/EV if any method of testing resulted positive. Baseline characteristics and clinical features were gathered via subject interviews and medical record abstractions. Variables were compared between subjects with RV/EV and two control groups: those negative for all pathogens and those negative for only RV/EV. Participants with RV/EV who had co-infections were excluded from the analysis. Descriptive statistics were performed using SAS v9.4.

**Results:**

Of 1429 enrolled participants, 123 (8.6%) were positive for RV/EV, of whom 111 had RV/EV alone. When compared to those negative for all tested pathogens (n=1034), participants with RV/EV more commonly had underlying COPD (45.0% vs. 35.5%, P=0.047) and less commonly had CHF (36.0% vs. 48.3%, P=0.014) or experienced acute myocardial dysfunction (29.7% vs. 41.2%, P=0.019). Participants with RV/EV also more commonly experienced fever (39.6% vs. 27.7%, P=0.008), cough (90.1% vs. 69.0%, P< 0.001), sore throat (54.1% vs. 39.5%, P=0.003), chest pain (48.6% vs. 37.8%, P=0.026), and dyspnea/respiratory distress (25.2% vs. 13.1%, P< 0.001) than those negative for all pathogens. Differences between RV/EV positive and negative groups were similar to the all pathogen negative group, with the exception of no significant differences in acute myocardial dysfunction, fever, and COPD in the RV/EV negative group.

**Figure:**
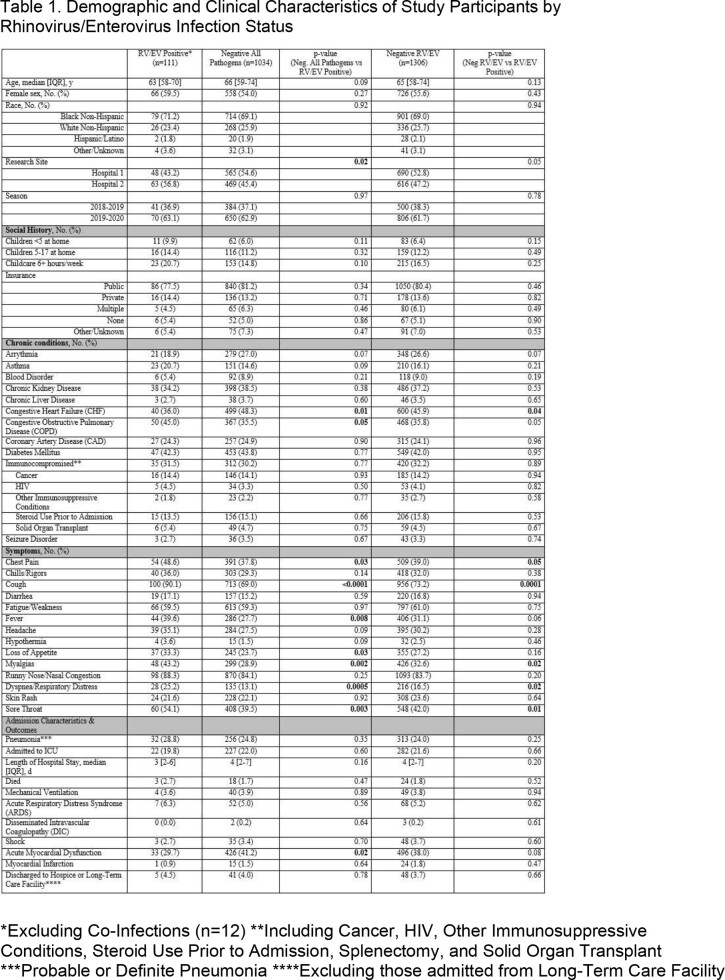

**Conclusion:**

Among older adults hospitalized with ARIs, CHF, and/or COPD exacerbations, RV/EV was associated with symptoms of both upper and lower respiratory tract infection and was more frequent identified among those with COPD.

**Disclosures:**

**Elizabeth Begier, M.D., M.P.H.**, Pfizer: EB is an employee of Pfizer, the sponsor of this study|Pfizer: Stocks/Bonds **Robin Hubler, MS**, Pfizer, Inc.: Employee|Pfizer, Inc.: Stocks/Bonds **Qing Liu, M.S.**, Pfizer Inc.: Stocks/Bonds **Bradford D. Gessner, M.D., M.P.H.**, Pfizer: I am an employee of Pfizer|Pfizer: Stocks/Bonds **Benjamin Lopman, PhD**, Epidemiological Research and Methods, LLC: Advisor/Consultant|Hillevax, Inc: Advisor/Consultant **Satoshi Kamidani, MD**, CDC: Grant/Research Support|Emergent BioSolutions: Grant/Research Support|NIH: Grant/Research Support|Pfizer Inc: Grant/Research Support **Nadine Rouphael, MD**, Icon, EMMES, Sanofi, Seqirus, Moderna: Advisor/Consultant **Evan J. Anderson, MD**, GSK: Advisor/Consultant|GSK: Grant/Research Support|Janssen: Advisor/Consultant|Janssen: Grant/Research Support|Kentucky Bioprocessing, Inc.: Safety Monitoring Board|Moderna: Advisor/Consultant|Moderna: Grant/Research Support|Moderna: Currently an employee|Moderna: Stocks/Bonds|Pfizer: Advisor/Consultant|Pfizer: Grant/Research Support|Sanofi Pasteur: Advisor/Consultant|Sanofi Pasteur: Grant/Research Support|Sanofi Pasteur: Safety Monitoring Board|WCG/ACI Clinical: Data Adjudication Board **Christina A. Rostad, MD**, BioFire Inc.: Grant/Research Support|GlaxoSmithKline Biologicals: Grant/Research Support|Janssen: Grant/Research Support|MedImmune LLC: Grant/Research Support|Meissa Vaccines, Inc.: RSV vaccine technology|Merck & Co., Inc.: Grant/Research Support|Micron Technology, Inc.: Grant/Research Support|Moderna, Inc.: Grant/Research Support|Novavax: Grant/Research Support|PaxVax: Grant/Research Support|Pfizer, Inc.: Grant/Research Support|Regeneron: Grant/Research Support|Sanofi Pasteur: Grant/Research Support

